# Revving up Natural Killer Cells and Cytokine-Induced Killer Cells Against Hematological Malignancies

**DOI:** 10.3389/fimmu.2015.00230

**Published:** 2015-05-13

**Authors:** Gianfranco Pittari, Perla Filippini, Giusy Gentilcore, Jean-Charles Grivel, Sergio Rutella

**Affiliations:** ^1^Department of Medical Oncology, National Center for Cancer Care and Research, Hamad Medical Corporation, Doha, Qatar; ^2^Deep Immunophenotyping Core, Division of Translational Medicine, Sidra Medical and Research Center, Doha, Qatar; ^3^Clinical Research Center, Division of Translational Medicine, Sidra Medical and Research Center, Doha, Qatar

**Keywords:** natural killer cell, cytokine-induced killer cell, interleukin-2, interleukin-15, good manufacturing practice, leukemia, immunotherapy

## Abstract

Natural killer (NK) cells belong to innate immunity and exhibit cytolytic activity against infectious pathogens and tumor cells. NK-cell function is finely tuned by receptors that transduce inhibitory or activating signals, such as killer immunoglobulin-like receptors, NK Group 2 member D (NKG2D), NKG2A/CD94, NKp46, and others, and recognize both foreign and self-antigens expressed by NK-susceptible targets. Recent insights into NK-cell developmental intermediates have translated into a more accurate definition of culture conditions for the *in vitro* generation and propagation of human NK cells. In this respect, interleukin (IL)-15 and IL-21 are instrumental in driving NK-cell differentiation and maturation, and hold great promise for the design of optimal NK-cell culture protocols. Cytokine-induced killer (CIK) cells possess phenotypic and functional hallmarks of both T cells and NK cells. Similar to T cells, they express CD3 and are expandable in culture, while not requiring functional priming for *in vivo* activity, like NK cells. CIK cells may offer some advantages over other cell therapy products, including ease of *in vitro* propagation and no need for exogenous administration of IL-2 for *in vivo* priming. NK cells and CIK cells can be expanded using a variety of clinical-grade approaches, before their infusion into patients with cancer. Herein, we discuss GMP-compliant strategies to isolate and expand human NK and CIK cells for immunotherapy purposes, focusing on clinical trials of adoptive transfer to patients with hematological malignancies.

## Biological Features of NK, LAK, and CIK Cells

Natural killer (NK) cells comprise 5–25% of peripheral blood (PB) lymphocytes and were initially recognized for their ability to kill cancer cells without prior sensitization. The reader is referred to previously published papers for a thorough review of NK development and function (1–3). Briefly, NK cells originate from bone marrow (BM) CD34^+^ hematopoietic stem cells and can also be differentiated *in vitro* from highly immature CD34^−^ umbilical cord blood (UCB) cells (4). NK cells acquire function (killing or cytokine production) after encountering and recognizing self-human leukocyte antigen (HLA) molecules during a process termed “licensing” or NK-cell education. However, 10–20% of NK cells remain unlicensed, as they lack receptors for self-major histocompatibility complex (MHC) and are functionally hyporesponsive. Importantly, unlicensed NK cells can become alloreactive upon encounter with cytokines in a recipient environment, e.g., after adoptive transfer into hematopoietic stem cell transplantation (HSCT) recipients.

The function of NK cells is governed by a set of germline-encoded activating or inhibitory receptors referred to as killer immunoglobulin-like receptors (KIRs). The extracellular domain determines which HLA class I molecule NK cells recognize, whereas the intracytoplasmic domain transmits either an activating or an inhibitory signal. KIRs are monomeric receptors with either 2 (KIR2D) or 3 (KIR3D) immunoglobulin-like domains, and are further subdivided into those with long (L) cytoplasmic tails (KIR2DL and KIR3DL) and short (S) cytoplasmic tails (KIR2DS and KIR3DS) (5–7). Long-tail KIRs generate an inhibitory signal through the recruitment of the SH2-domain-containing tyrosine phosphatase 1 protein (SHP1) (8–11). Short-tail KIRs possess truncated portions that transduce activating signals via tyrosine phosphorylation of DAP12 and other proteins (12–14).

Natural killer cells also express other activating receptors that recognize “stress ligands” on virally infected or malignant cells. For instance, NKG2D, a C-type lectin receptor that belongs to the NK group 2 (NKG2) of receptors as member D (15), is constitutively expressed on NK cells and recognizes MHC class I chain-related genes A and B (MICA and MICB) (16), as well as unique long 16 (UL16) binding protein family members (ULBPs) (17). Other activating molecules include natural cytotoxicity receptors (NCRs) NKp30, NKp44, and NKp46 (18, 19). It has been shown that killing of tumors of non-epithelial origin, including leukemia cell lines, involves synergism between NCRs and NKG2D (20). Activating KIRs, such as KIR2DS1, are likely involved in the anti-leukemia effect of NK cells (21, 22). In 2002, investigators from Perugia demonstrated superior disease-free survival (DFS) in patients with acute myeloid leukemia (AML) receiving BM grafts from HLA-haploidentical donors who expressed KIR binding to MHC class I molecules absent in the host (i.e., KIR-ligand mismatch in the GVH direction) (23, 24). The most notable inhibitory receptors recognize HLA class I proteins (including groups of HLA-A, HLA-B, and HLA-C) and differ in both their transmembrane and intracytoplasmic domains (25–29).

Human leukocyte antigen-C is the predominant class I isotype involved in the inhibitory and activating regulation of human NK cells (1, 22). Individuals may have up to 15 KIR genes that reside in a single complex on chromosome 19p13.4. KIR genes can be divided into A or B haplotypes. The A haplotype consists of five inhibitory KIRs and a single activating KIR, KIR2DS4. By contrast, the B haplotype contains both inhibitory and several activating KIRs that are further subdivided into two separate regions, centromeric and telomeric. In the “missing self” model (30), donor NK cells express inhibitory KIRs for which HLA class I molecules are missing in the recipient. Donors with KIR B vs. KIR A haplotypes improve the clinical outcome for patients with AML by reducing the incidence of leukemia relapse and prolonging DFS (31). The centromeric KIR B genes were dominant over the telomeric ones, and included the genes encoding inhibitory KIRs that are specific for the C1 and C2 epitopes of HLA-C. When the authors examined a cohort of 1,532 T-cell-replete HSCT, relapse protection associated with donor KIR B was enhanced in recipients with one or two C1-bearing HLA-C allotypes compared with homozygous recipients. This implies that a deeper understanding of the interaction between donor KIRs and recipient HLA class I will allow the selection of the “best donor” to improve outcomes of unrelated HSCT and adoptive NK infusion for AML. Intriguingly, KIR B haplotype donors were recently shown to confer a reduced risk for relapse after haploidentical HSCT in children with ALL (32), an effect that is not seen in adult ALL (33). In allogeneic HSCT, particularly from HLA-mismatched donors, NK cells reportedly influence clinical outcome by exerting anti-tumor effects without inducing graft-versus-host disease (GVHD) (34). However, NK cells reconstituting after allogeneic HSCT may be dysfunctional, likely as a result of low IL-2 levels (35). Some groups are attempting to improve NK-cell reconstitution following HSCT by depleting the graft of αβ^+^ T cells and CD19^+^ B cells, but leaving NK progenitors untouched (36). Using this approach, very high numbers of haploidentical NK cells and NK-like (CD56^+^) T cells can be infused into patients with malignant disorders (37).

Another family of human NK receptors is composed of a common subunit (CD94), covalently linked to a distinct chain encoded by a C-type lectin NKG2 family gene. Among the C-type lectin NK receptors, CD94/NKG2A is inhibitory, whereas other heterodimers are activating receptors. CD94/NKG2A binds the non-classical class I molecule HLA-E (38). The binding of a unique peptide/HLA-E complex to the activating CD94/NKG2C receptor is of higher affinity than the binding to the inhibitory CD94/NKG2A ensuring the predominance of inhibitory signals when the same NK cells express both activating and inhibitory receptors recognizing HLA molecules (39).

In 1980, Rosenberg and co-workers demonstrated that incubation of heterogeneous lymphocyte populations with high-dose (800-1,000 U/ml) interleukin-2 (IL-2) generates lymphokine-activated killer (LAK) cells with prompt *in vitro* cytotoxicity to syngeneic and autologous fresh tumors (40–42). NK cells were identified as precursors of LAK cells, and LAK activity was found to be mainly, albeit not uniquely, mediated by activated NK cells (43, 44). LAK cells comprise CD3^−^CD56^+^ NK cells, MHC-unrestricted cytotoxic CD3^+^CD56^+^ T cells, and CD3^+^CD56^−^ T cells. However, LAK cells had limited expansion *in vitro* and low cytolytic activity *in vivo*. Furthermore, LAK therapy required high doses of IL-2 *in vivo* and was associated with relevant toxicity. Modifications in culture conditions, i.e., provision of agonistic αCD3 (OKT3) monoclonal antibodies (mAbs), IL-2 and interferon (IFN)-γ, translated into >1,000-fold expansion of peripheral blood mononuclear cells (PBMCs) with potent cytokine-induced killer (CIK) activity. CIK cells share phenotypic and functional properties of both T cells and NK cells, as they co-express CD3 and CD56, and are rapidly expandable in culture like T cells, while not necessitating functional priming for *in vivo* activity, analogous to NK cells. Interestingly, CIK cells do not recognize target cells through the T-cell receptor (TCR) and do not require the presence of MHC molecules on target cells, as suggested by the observation that cytotoxicity is not affected by antibody masking of the TCR or MHC class I or class II molecules. CIK cells also express activating NK receptors, including NKG2D, DNAX accessory molecule-1 (DNAM), and NKp30 (45, 46).

Evidence for an *in vivo* activity of CIK cells derives from studies in a murine severe combined immune deficiency (SCID)/human lymphoma model, where co-administration of CIK cells with B-lymphoma cells had favorable effects on mice survival, with a 1.5–2.0-log cell kill and only marginal toxicity against normal hematopoietic precursors (47). CIK cells reportedly protect against syngeneic and allogeneic tumors also in other experimental models, including nude mice xenografted with human cervical carcinoma cells (48–50). CIK cells are detected in the lungs 30 min after injection, followed by distribution to other sites, such as the liver and spleen and, by 72 h, the tumor site, where CIK cells may remain for more than 9 days (51).

## Current NK-Cell Manufacturing Practices

A direct comparison of NK manufacturing techniques is hampered by differences in starting materials, technologies, and manipulation strategies (52, 53). Classically, GMP-compliant NK-cell products have been generated from PBMCs collected by apheresis (Table 1). It has been shown that NK cells obtained from granulocyte colony-stimulating factor (G-CSF)-mobilized leukapheresis products have reduced functional capacity (54). Conceivably, non-mobilized blood may be preferable over G-CSF mobilized blood as a source of NK cells for immunotherapy trials. A variety of cellular media have been used to culture NK cells, including X-VIVO serum-free medium, AIM V, or stem cell growth medium (SCGM), typically supplemented with 5–10% human AB serum to enhance NK function. Because the limited number of NK cells in leukapheresis products restricts clinical applicability, *in vitro* methods to expand NK cells are intensely being developed. In this respect, IL-15 promotes NK-cell proliferation and survival, and has been variably used in GMP-grade laboratory protocols, as further detailed below. Alternative methods of expansion rely on human feeder cells, including artificial antigen presenting cells (APCs) that are modified with costimulatory molecules, such as CD137 ligand, and membrane-bound (mb) IL-15 or IL-21. However, expanded NK cells undergo exhaustion, as shown by telomere shortening and replicative senescence.

**Table 1:** **Current GMP-compliant NK-cell manufacturing methods are detailed**.

Reference(s)	Cells	Manufacturing process	Feeder cells	Characteristics	Purity
(55)	UCB	CD34 immunoselection; expansion in a bioreactor (SCF, Flt3-L, TPO or IL-15, IL-7, G-CSF, GM-CSF, and IL-6 from d0 to d14; same as above +IL-2 from d14 onward)	Not used	≈2,100-fold expansion; 1.6–3.7 × 10^9^ NK cells; undetectable T and B cells	90–95% NK cells
(56)	UCB	IL-15, IL-2, OKT3, and heparin, with or without tacrolimus	Not used	1,700-fold expansion; ≈40 × 10^6^ NK cells from 1.0 × 10^6^ UCB cells	>70% NK cells
(57)	PBMCs; LK	CD3 depletion; overnight incubation with IL-2	Not used	<5 × 10^6^ residual T cells; >70% viability	>18% NK cells
(58)	PBMCs; LK	CD3 depletion (protocol I); CD56 enrichment (protocol II); overnight incubation with IL-2	Not used	686.7 × 10^6^ and 253.2 × 10^6^ NK cells with protocols I and II, respectively	38% (I) and 90% NK cells (II)
(59)	PBMCs; LK	CD3 depletion; CD56 enrichment; no exposure to IL-2 or other cytokines	Not used	Median of 29 × 10^6^ NK cells/kg infused	0.097 × 10^6^/kg contaminating B cells; 1 × 10^3^/kg T cells in 1 product
(60)	PBMCs; LK	CD3 depletion; CD56 enrichment	Not used	1.1–8.8 × 10^8^ NK cells	<0.01% T cells
(61)	PBMCs; LK	IL-2+ anti-OKT3 in various flasks, culture bags, and bioreactors for 20 d	Not used	530 to 1,100-fold NK-cell expansion	31–51% NK cells
(62)	PBMCs; LK	CD3/CD19/CD4/CD33 depletion; incubation with IL-2 and IL-15 for 7–21 d	Irradiated autologous PBMCs	100-fold NK-cell expansion at 16 d	91% CD56^+^ cells
(63)	NK-92 cells from a master cell bank	IL-2	Not used	2.0–42.4 × 10^9^ (starting from 5 × 10^7^)	N.A.
(64)	NK-92 cells from a master cell bank	IL-2 for 15–17 d	Not used	>200-fold NK-cell expansion	1.5 × 10^9^ cells/L
(65)	PBMCs; LK	CD3 depletion; CD56 enrichment; overnight incubation with IL-2, OKT3, with or without IL-15	Irradiated autologous PBMCs	62.7-fold NK-cell expansion	
(66)	PBMCs; LK	CD56 enrichment; overnight incubation with IL-2, with or without IL-15, for 14 d	Not used		67% NK cells
(67)	PBMCs; LK	CD3 depletion; IL-2 for 7 d	K562–mb15–41BBL		73% NK cells with <14% T cells
(68)	Autologous PBMCs; LK	CD3 depletion; IL-2 and OKT3 for 21 d	Irradiated autologous PBMCs	1.88–7.6 × 10^10^ NK cells	>93% purity
(69)	Autologous PBMCs; LK	CD3 depletion; CD56 enrichment; IL-2 for 28 d	Irradiated EBV-TM-LCLs	>96% NK cells, with no CD3^+^ T cells	
(70)	PBMCs; LK (1-h)	CD56 selection (Clini-MACS^®^; research-grade); CD3 depletion (Dyna Beads^®^; research-grade); partially automated separation procedure, clean-room conditions (“class A in B”)	Not used	160 × 10^6^ NK cells (<0.01% remaining CD3^+^ T cells)	98.6% purity
(71)	Autologous PBMCs from patients with MM	SCGM with IL-2 and OKT3	Not used	1,625-fold NK-cell expansion on d20	65% NK cells
(72)	PBMCs; LK	CD34 selection (Clini-MACS^®^); culture with research-grade SCF, Flt3-L, IL-7, and hydrocortisone for 21 d and with research-grade IL-15, IL-21, and hydrocortisone for additional 21d	Not used	9.28 × 10^6^/kg NK cells from 2.2 × 10^6^/kg CD34^+^ cells	64% NK cells with 1% CD3^+^ T cells
(73)	PBMCs; LK	CD3 depletion; CD56 selection	Not used	12.1 × 10^6^/kg NK cells	0.03 × 10^5^ median T-cell dose
(74)	PBMCs; LK	CD3 depletion; overnight IL-2 in four procedures, IL-2 during exposure to CD3 beads in six procedures	Not used	>1.0 × 10^6^/kg NK cells	<1.0 × 10^5^/kg T cells
(75)	PBMCs; LK	CD3 depletion; CD56 selection	Not used	5.0 × 10^6^/kg NK cells in 77% of patients	93.5% purity
(76)	PBMCs; LK	CD3 depletion; IL-2 for 8–16 h	Not used	21.0 × 10^6^/kg NK cells	43% purity
(77)	PBMCs; LK	NK-cell priming with CNDO-109 lysate (derived from a leukemia cell line, CTV-1)	Not used	<10^4^ residual T cells	
(78)	PBMCs; LK	CD3 depletion; 1,000 U/ml IL-2	Not used	Median 26% haploidentical NK cells; three dose levels	Median viability >95%

*PBMC, peripheral blood mononuclear cells; LK, leukapheresis; d, day; UCB, umbilical cord blood; SCGM, stem cell growth medium; MM, multiple myeloma*.

In 2001, Carlens and co-workers described a cytokine-based technique for *in vitro* enrichment of human NK cells from bulk PBMCs of healthy individuals (79). PBMCs were incubated in SCGM containing 5% human serum and varying concentrations of IL-2. In addition, stimulation with OKT3 at 10 ng/ml was provided during the first 5 days of the culture. Supplementation with 500 U/ml IL-2 yielded a median 193-fold cell expansion in 21 days. Fifty-five percent of the expanded cells had a CD3^−^CD56^+^ phenotype, and prolongation of the culture beyond 3 weeks did not allow further NK-cell enrichment. Moreover, expansion of the NK-cell compartment was comparable in cultures containing IL-2 concentrations ranging from 100 to 1,000 U/ml. Expanded cells could efficiently kill the NK-susceptible K562 line. This protocol was subsequently applied to PBMCs from patients with multiple myeloma (MM), an incurable plasma cell malignancy with a unique ability to subvert anti-tumor immune responses (80). Following an initial non-proliferative phase of 5 days, patient-derived NK cells expanded 1,625-fold on average after 20 days of culture (71). NK cells from MM patients displayed increased expression of multiple activating receptors, including 2B4, NKp46, NKp44, NKp30, NKG2D, and DNAM-1, and were efficiently cytotoxic to K562 cells and primary autologous MM cells, but not to autologous CD34^+^ cells (71). Mobilized PBMCs from patients with MM have also been used to expand NK cells (81). After a 7-day culture with serum-free AIM V media, IL-2 and OKT3, polyclonal populations of cytotoxic lymphocytes were detected, including CD4^+^ T cells, CD8^+^ T cells, CD8^+^CD56^+^ T cells, and CD56^+^ NK cells. Culture bags provided a two- to threefold expansion of immune effectors that retained their cytotoxicity after cryopreservation and thawing.

Notably, *ex vivo* expansion of NK cells from PBMCs incubated with IL-2 was also pursued under GMP-compliant conditions. Using an automated bioreactor system, bulk PBMCs from healthy donors and MM patients could expand 77-fold on average, and acquired enhanced cytotoxicity that positively correlated with the up-regulation of the NKp44 activating receptor. However, the expanded culture contained a significant proportion of T cells, necessitating further T-cell depletion prior to clinical use (61). Furthermore, purified CD56^+^ populations were positively selected from PBMCs of healthy individuals using CD56 magnetic microbeads, and cultured in X-VIVO 10 medium containing 10% human AB serum and 500 U/ml IL-2 ± 10 ng/ml IL-15 for 2 weeks. Appreciable proliferation occurred 5–7 days from the start of the culture, although with remarkable donor-to-donor variability. Expansion of CD3^+^CD56^+^ NK-like T cells was two to three times greater than that of CD3^−^CD56^+^ NK cells and was not affected by IL-15. Compared with the NK-92 cell line, *ex vivo* expanded CD56^+^ cells had lower lytic activity against both K562 and Raji target cells (66).

The natural nicotinamide adenine dinucleotide (NAD)^+^ precursor and NAD^+^-dependent enzyme inhibitor nicotinamide (NAM) has been recently shown to induce a 60- to 80-fold NK-cell expansion when added to feeder-free cultures containing IL-2 and IL-15 (82). In this study, NAM also affected NK cell anti-tumor capabilities and trafficking properties by modulating expression of CD200R and PD1, two immune regulatory receptors that transmit inhibitory signals upon interaction with cognate ligands on cancer cells. In addition, NAM promoted surface expression of L-selectin, an adhesion molecule mediating interactions with vascular endothelium and lymph nodes.

### CD3^+^ T-cell depletion with or without CD56 enrichment

The CE-approved, partially automated Clini-MACS^®^instrument from Miltenyi allows the enrichment of NK cells under GMP-compliant conditions (58). After a single step of magnetic CD3^+^ T-cell depletion, PBMCs are stimulated and expanded with irradiated autologous cells in the presence of OKT3 and IL-2, resulting in a highly pure population of functional CD3^−^CD16^+^CD56^+^ NK cells that lack cytotoxicity against allogeneic non-tumor cells (83) (Table 1). Immunomagnetic CD3^+^ T-cell depletion with either the 2.1 or the 3.1 programs can be combined with CD56-cell enrichment (84). When CD56^+^ cells are magnetically isolated, the expansion of CD3^+^CD56^+^ cells in culture may outweigh that of CD3^−^CD56^+^ cells, since CD3^+^ cells are not depleted upfront (66). Furthermore, CD56 expansion in cultures supplemented with IL-2, either alone or in combination with IL-15, shows substantial inter-donor variability. Each of the above programs translates into differences in depletion efficiency and recovery of NK cells, with NK purification being improved after sequential processing with the Clini-MACS T-cell depletion programs D2.1 and D3.1. Not unexpectedly, absolute NK-cell numbers after manipulation may correlate with the pre-harvest NK-cell content of the PB (85), implying that donors with high NK-cell counts are likely to provide NK-cell products with the highest cell numbers. A clinical-scale procedure to isolate NK cells for infusion in pediatric patients was developed under clean-room conditions (70). One-hour leukapheresis collections from unstimulated healthy donors were used to positively select CD56^+^ cells and negatively deplete T cells, ultimately leading to cell therapy products enriched in NK cells and containing only 0.09% remaining T cells. A similar procedure consisting of two rounds of CD3 depletion and one round of CD56 selection has been used to obtain clinically applicable numbers of NK cells for immunotherapy (86). In that study, NK cells were expanded with IL-2 for 10–14 days to achieve the desired cell dose for potential clinical application in three children with relapsed or refractory leukemia after haploidentical HSCT.

Natural killer cells can also be expanded with irradiated autologous feeder cells, IL-2, IL-15, and anti-CD3 antibodies. Using these systems, NK cells acquire a CD56^int^CD16^int^ phenotype and increase an average of 117-fold in 3 weeks (65). IL-2 and IL-15 mediate better NK expansion and viability compared with cultures nurtured with IL-2 only. Importantly, the number of residual contaminating T cells may be significantly lower after NK-cell exposure to IL-2 and IL-15 compared with IL-2 alone. NK cells activated with IL-2 and IL-15 may display higher cytotoxicity against K562 cells when kept in culture at a low effector-to-target ratio (66). In order to selectively expand alloreactive NK cells, KIR^+^ cells can be isolated from Clini-MACS-purified CD3^−^CD56^+^ NK cells using cell sorting, and then stimulated with the same cytokine cocktail (65). GMP-sorted and expanded single KIR^+^ cells were cytolytic against AML blasts, an effect that was more pronounced than that mediated by bulk NK cells in an HLA-mismatched setting.

Interleukin-21 can offer theoretical advantages for the expansion of NK cells. The temporal exposure of IL-2/IL-15-stimulated NK cells to IL-21 determines the extent to which NK-cell proliferation and function are promoted (87). Specifically, NK cells stimulated with IL-21 during the first week of culture were shown to have strong proliferative response and cytotoxic activity compared with control cultures. The short-term expanded NK cells had longer telomeres than NK cells maintained with IL-21 continuously. IL-21 has also been used in combination with IL-15 to activate HLA-mismatched NK cells derived from CD34^+^ hematopoietic progenitors with SCF, Flt3-L, IL-15, and hydrocortisone (72).

### Use of feeder cells

While the minimum necessary NK-cell number for therapeutic efficacy is still controversial, the consistent generation of large amounts of functional cells is crucial to develop clinical protocols of adoptively transferred NK cells. Different feeder cell types have been used to expand NK cells, including irradiated PBMCs, EBV-transformed lymphoblastoid cell lines (EBV-LCL), gene-modified K562 cells expressing NK cell-stimulatory molecules such as 41BB-ligand and mbIL-15 (67). Compared with IL-2-mediated activation, NK-cell expansion in the presence of feeder cells may also result in increased anti-tumor cytotoxic functions, with comparable *in vivo* survival (69, 88).

K562 cells were transduced with constructs encoding mbIL-15 (IL-15 + CD8α) and human 41BB-ligand (both containing green fluorescent protein). NK-cell recovery was 21.6-fold after 7 days of culture and increased to 152-fold and 277-fold after 14 and 21 days of culture, respectively. Importantly, the median recovery of NK cells was comparable when mononuclear cells from patients with acute leukemia were used in the co-culture. The expanded NK cells were cytotoxic against both AML cell lines and primary AML blasts. When compared with IL-2-stimulated NK cells, the cytotoxicity of expanded NK cells was greater at all effector-to-target ratios (67). In a mouse model of AML, multiple injections of expanded NK cells vigorously suppressed leukemia growth, with some mice achieving long-term control of the disease in the absence of xenogeneic GVHD. Finally, a master cell bank of K562–mb15–41BBL cells was established following GMP guidelines. The transduced NK cells were used to expand NK cells from leukapheresis collections at a 1:10 NK cell-transduced K562 cell ratio. The expansion of NK cells ranged from 33- to 141-fold after 7 days in culture. The overall yield of NK cells was higher than that observed in small-scale experiments.

A GMP-compliant NK-expansion methodology was also applied to patients with metastatic melanoma or renal cell carcinoma. A 278- to 1,097-fold NK-cell expansion was obtained when OKT3-loaded, 30-Gy-irradiated autologous PBMCs were used as feeders in AIMV medium containing 10% human AB serum and 600 U/ml IL-2 for 21–26 days. Following adoptive transfer to patients treated with a lymphodepleting regimen, expanded NK cells persisted for multiple days, likely representing the majority of NK cells in the circulation 1 week after infusion (68). Autologous PBMCs have also been used as feeders for the expansion of NK cells from healthy donors. Feeder cells obtained from the NK-depleted fraction of donor leukapheresis collections were used at a 10:1 feeder/NK-cell ratio for a GMP-compliant expansion procedure in Baxter LifeCell culture bags containing SCGM CellGro medium, 5% human AB serum, and 200 U/ml IL-2 with or without IL-15 supplementation. This protocol was successful in propagating cultured NK cells, which expanded 117 ± 20-fold after 19 days in the presence of 10 ng/ml IL-15 (65). More recently, a similar NK-cell expansion efficiency was reported when NK cells from healthy donors or patients with ALL in CR were co-cultured with autologous PBMCs in CellGro SCGM medium containing IL-2 and IL-15 (respectively, 34.9- vs. 39.5-fold average expansion after 14 days) (89).

Allogeneic PBMCs have been used as feeder cells for large-scale expansion of clinical-grade NK cells (62, 69). Allogeneic PBMCs and NK cells were co-cultured in X-VIVO 20 medium containing 500 U/ml IL-2 (69), or 100 U/ml IL-2 and 10 ng/ml IL-15 (62). In these studies, a similar 80- to 100-fold NK-cell expansion was achieved in 14–15 days. In an interesting study from Kim and colleagues, autologous PBMC feeders from cancer patients or PBMCs from healthy donors were compared (90). Co-cultures containing PBMCs from healthy donors could more efficiently propagate NK cells than those containing PBMCs from cancer patients (respectively, 300- vs. 169.4-fold average expansion after 14 days).

**Figure 1:**
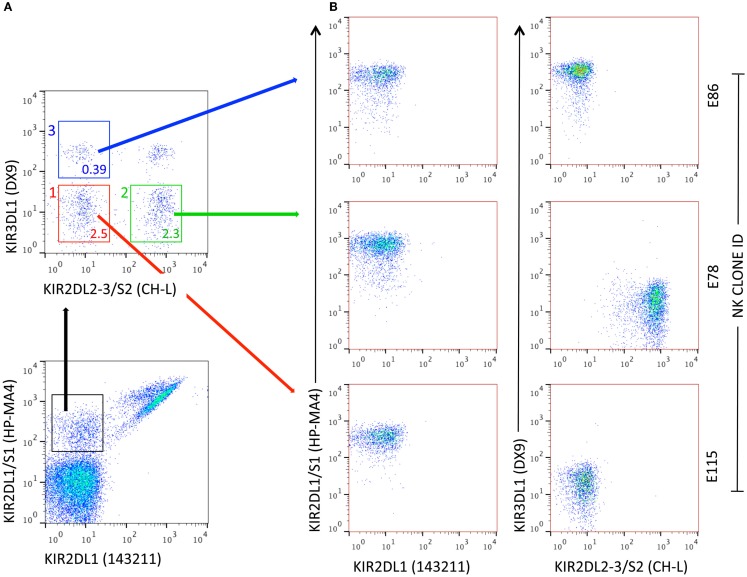
**Generation of NK clones from individual NK cells with specific KIR receptor repertoires**. **(A)** Flow cytometry representation of NK-cell subsets defined by a combination of four anti-KIR mAbs. In this example, subsets used for FACS-assisted single-cell deposition express 2DS1, either alone (subset 1, red) or in combination with at least one receptor among 2DL2, L3, and S2 (subset 2, green) or 3DL1 (subset 3, blue). The percent frequency of NK-cell subsets is indicated. **(B)** Representative NK clones obtained after 3-week *in vitro* propagation in the presence of IL-15 *trans*-presentation. E115: 2DS1^pos^; E78: 2DS1^pos^/CH-L^pos^; E86: 2DS1^pos^/3DL1^pos^. For E78, specific KIR(s) can be identified by real-time RT-qPCR.

Pittari and colleagues described a novel technique for selection, deposition, and high-efficiency cloning of individual NK cells displaying surface receptor repertoires of choice. Cells were selected by FACS, deposited into *U*-shaped polystyrene 96-well plates (one cell per well) containing CellGro SCGM medium supplemented with 10% human AB serum and without exogenous cytokines. Propagation of NK clones from single cells was driven by *trans*-presentation of IL-15 by BaF/3 pre-B-lymphocytes double transfected with human IL-15Rα and human IL-15 (BaF/3 IL-15Rα/IL-15). Additional feeder cells were EBV-BLCL (JY) and PBMCs from three allogeneic donors (91). In this pre-clinical design, the technique allowed for prompt propagation of NK clones from NK-cell populations potentially involved in the control of leukemia relapse, i.e., expressing the KIR2DS1 activating receptor (22), regardless of their frequency (Figure 1). After 3 weeks, propagated NK clones typically reached 0.25–4 × 10^6^ cells, with an overall cloning efficiency as high as 35–40%.

The replicative potential of NK cells expanded with genetically modified K562 cells can be further enhanced by enforcing the expression of human telomerase reverse transcriptase (TERT) gene (92). After stimulation with K562 cells for 1 week, NK cells were transfected with a retroviral vector containing human *TERT*. At variance with the control cultures that underwent replicative senescence after 16 population doublings, TERT-NK cells continued to expand *in vitro* for more than 1,000 days, if periodically re-stimulated with K562 cells. However, NK cells accumulated genetic changes at late time-points, including gain in genes on chromosome 1 and losses in genes on chromosome 16, suggesting that genetic instability may be a limiting factor in immortalization of NK cells.

Gas-permeable cell culture devices (G-Rex) are being evaluated for the expansion of T cells and tumor cells. In these systems, gas exchange across the base of the culture allows increased volumes of medium per unit area, augments the rate of cell expansion, and decreases cell death, minimizing cell manipulation. Using this strategy, up to 19 × 10^9^ functional NK cells were produced starting from leukapheresis products, within 8–10 days of culture (93). The contaminating T cells mostly comprised CD8^+^ T cells and could be removed by magnetic depletion. When compared with conventional gas-permeable bags, the G-Rex yielded higher fold expansions of NK cells, requiring no *interim* manipulation or feeding during the culture period. The NK cells were viable and functional, even after 12 months of cryopreservation.

### Use of cord blood and other stem cell sources to expand NK cells

Umbilical cord blood is an emerging source of NK cells for clinical applications and also provides an *in vitro* system to analyze NK development (4). Banked UCB units represent an ideal “off-the-shelf” source of NK cells for adoptive immunotherapy. Importantly, NK cells from PB and UCB differentially express cytokine receptors, with IL-15Rα being preferentially detected on UCB NK cells and IL-12Rβ1 and IL-18α receptors being primarily found on PB NK cells (94, 95). The combination of IL-15 and IL-18 optimally stimulates the proliferation of UCB NK cells and potentiates the release of IFN-γ and TNF-α. The lower responsiveness of UCB NK cells to IL-2 observed in these studies may be the result of lower expression of IL-2 receptors and of decreased phosphorylation of STAT5 as compared with PB NK cells. This implies that, at variance with PB NK cells that are fully activated by IL-2 alone, UCB NK cells may require additional cytokine stimuli (96). For instance, the addition of tacrolimus and low-molecular-weight heparin significantly enhances NK-cell expansion induced by IL-2, IL-15, and anti-CD3 mAbs (56). Using this protocol, approximately 40 × 10^6^ NK cells were obtained from 1 × 10^6^ unmanipulated UCB cells, in the absence of feeder cells, corresponding to >1,000-fold expansion. Bioreactors have been used to expand UCB-derived NK cells as well. This approach resulted into the generation of a clinically relevant dose of NK cells with >2,000-fold expansion, purity of >90%, high expression of activating receptors and cytolytic activity against K562 leukemia cells (55).

It has been shown that UCB-derived NK cells actively migrate to the BM, spleen, and liver 24 h after infusion in NOD-SCID-IL-2Rγ-null mice (97). NK cells were differentiated in 3–4 weeks from CD34^+^ hematopoietic progenitors exposed to multiple cytokines, and were found to express CXCR4, CXCR3, and CCR6, which likely accounted for their ability to home to BM and inflamed tissues. A single NK-cell infusion combined with *in vivo* low-dose IL-15 resulted in inhibition of leukemia growth and prolongation of mice survival.

Finally, human embryonic stem cells (ESCs) as well as induced pluripotent stem cells (iPSCs) are potential sources of phenotypically mature and functional NK cells (98). ESCs and iPSCs were first used to produce hematopoietic progenitors with the “spin embryonic body (EB)” method, in which defined numbers of cells were spin-aggregated in serum-free medium. This strategy removed the need for murine stromal support, and led to hematopoietic cell development and proliferation. Spin EB-derived cells were then tested in a feeder-free and serum-free system containing NK-cell promoting cytokines, i.e., IL-3, IL-7, IL-15, SCF, and Flt3-L. Within the first 2 weeks of culture, both non-adherent CD31^+^ endothelial cells and CD73^+^ mesenchymal stromal cells were detected. Importantly, NK cells developed in similar numbers, phenotype, and functional characteristics as those differentiated with the use of murine stromal cells (98). Artificial APCs engineered to express mbIL-21 additionally expanded NK cells. As the expected requirement for NK-cell adoptive transfer protocols is approximately 2 × 10^7^ NK cells/kg (see below), genetically modified APCs allow the use of a starting population of <10^6^ ESCs/iPSCs per patient, corresponding to a lower number of cells compared with that required for NK-cell expansion from the PB.

### Use of genetically engineered NK cells

Although lentiviral (LV) vectors have been successfully used to transduce both T cells and NK-cell lines, LV transduction of both freshly isolated and *ex vivo*-expanded NK cells may be challenging. Chimeric antigen receptors (CARs) are synthetic engineered receptors that target surface molecules in their native conformation, independent of MHC and of antigen processing by the target cells (99). The generations of CARs are typically classified based on the intracellular signaling domains, with first-generation CARs including only CD3ζ, second-generation CARs including one single costimulatory domain and third-generation CARs including two costimulatory domains, such as CD28 and 41BB.

Natural killer cells can be transduced with mRNA encoding for anti-CD19 CARs. The expression of a receptor containing CD3ζ and 41BB signaling molecules (anti-CD19-BB-ζ) was induced in human NK cells with a clinical-grade electroporator. The cytotoxicity of the transfected NK cells was evaluated both *in vitro* and in a mouse model of leukemia. Receptor expression was already detectable 6 h after electroporation, reaching maximum levels at 24–48 h. Toxicity against CD19-expressing targets was specifically observed at 96 h. Median anti-CD19-BB-ζ expression 24 h after electroporation was 40.3 and 61.3% in freshly purified and in expanded NK cells, respectively. NK cells expressing anti-CD19-BB-ζ secreted IFN-γ in response to CD19^+^ target cells and had enhanced cytotoxicity against B-cell malignancies (100). Transduced NK cells were consistently more cytotoxic than non-transduced NK cells. A large-scale, GMP-compliant protocol was also developed and showed that median percentage of genetically modified NK cells with receptor expression was 82% after 24 h. NK cells transfected under these conditions exerted *in vivo* cytotoxicity in NSG mice with B-cell leukemia, and suppressed leukemia progression compared with mice inoculated with mock-transfected NK cells (100). Interestingly, NK cells can acquire anti-CD19 CARs from donor cells via trogocytosis (101). When co-cultured with live K562 cells transduced to express anti-CD19-BB-ζ, NK cells acquired anti-CD19 CARs, peaking at 1 h and declining thereafter. NK cells displayed enhanced degranulation in response to leukemia cell lines compared with NK cells co-cultured with control cells.

Genetically modified NK-cell lines, such as NK-92 cells, have been tested for *in vitro* and *in vivo* efficacy against MM. IL-2-independent derivatives of NK-92 cells, i.e., NK-92MI cells, have been transduced with a first-generation CAR targeting CD138, an integral membrane protein expressed on differentiated plasma cells (102). Genetically modified NK-92MI cells harbored a CAR consisting of an anti-CD138 single-chain variable fragment (scFv) fused to CD3ζ chain. The retargeted NK cells (NK-92MI-scFv) released IFN-γ and granzyme-B, and lysed CD138-expressing MM cell lines. When assayed in a xenograft NOD-SCID mouse model, transduced NK cells exerted more potent anti-tumor activity toward CD138-expressing MM cells than NK-92MI-mock. Importantly, NK cells could be detected in the MM microenvironment more than 20 days after their adoptive transfer.

CS1 is another surface protein highly expressed on MM cells and is amenable to targeting with CS1-specific CARs (103). CS1 co-localizes with CD138 on polarized plasma cells where it promotes adhesion, clonogenic growth, and tumorigenicity. Compared with mock-transduced NK cells, CS1-CAR-transduced NK cells had increased cytotoxic activity against CS1-expressing MM cells and showed heightened IFN-γ production. In an orthotopic MM xenograft model, adoptively transferred CS1-CAR-NK-92 cells suppressed the growth of human IM9 MM cells and significantly prolonged mouse survival (103). Overall, studies with CAR-NK cells point to the efficacy of this approach. The safety profile of CAR-NK cells may be advantageous compared with that of CAR-T cells, because of lack of *in vivo* clonal expansion and cytokine storm. Also, CAR-NK cells should not induce GVHD, while potently mediating GVL effects.

Natural killer cells can also be transduced to express mbIL-15 (104). Compared with NK cells expressing wild-type IL-15, mbIL-15 NK cells secreted low amounts of IL-15 in culture supernatants. Membrane-bound IL-15 appeared to be mostly occupying autologous receptors, suggesting that mb-IL-15 preferentially stimulates cells in *cis*, i.e., by direct binding to receptors expressed in the same cell. Genetically modified NK cells were maintained and expanded in culture without exogenous IL-2. When tested *in vitro* and *in vivo*, mbIL-15 NK cells displayed enhanced survival and cytotoxicity, being capable of inhibiting the growth of AML and sarcoma cells in NOD-SCID IL-2Rγ-null mice.

A complementary approach to existing methods of genetic modification of NK cells is offered by a retroviral vector-based, gene transfer protocol (105). Using a SFα11GFP viral vector, transduced NK cells were visible as GFP-expressing cells by fluorescence microscopy. The median transduction efficiency after one or two rounds of transduction was 27 and 47%, respectively. On day 21 of culture, transduction efficiencies averaged 52 and 75%, respectively. The gene transfer procedure did not affect NK-cell phenotype or function, suggesting that retroviral vectors can be successfully applied to immunotherapy trials.

Clinical experience with CAR-engineered NK cells is in its infancy (106). Two clinical trials are currently open with the aim at exploring the therapeutic benefit of haploidentical NK cells modified with anti-CD19 CARs in children with B-cell precursor ALL (ClinicalTrials.gov NCT00995137) and in children and adults with refractory ALL (ClinicalTrials.gov NCT01974479). In these studies, NK cells will be expanded by co-culture with irradiated K562 cells modified to express mbIL-15 and 41BB-ligand. The expanded NK cells will be then transduced with a signaling receptor that binds to CD19 (anti-CD19-BB-ζ).

### Use of expanded NK-cell lines

Several malignant NK-cell lines have been established and used for clinical trials in China, Japan, and Western Countries, as reviewed elsewhere (107). A potential drawback of this approach is that differences in HLA molecules across different ethnicities may translate into the production of HLA-specific antibodies by the recipient. NK-92 cells, the most extensively characterized NK-cell line, were established in 1994 from the PB of a male Caucasian patient with non-Hodgkin lymphoma, are IL-2-dependent and harbor a CD2^+^CD56^+^CD57^+^ phenotype (108–112). The adoptive transfer of NK-cell lines has theoretical advantages related to lack of expression of inhibitory KIRs, lack of immunogenicity, and ease of expansion. The optimal conditions for large-scale *ex vivo* expansion of NK-92 cells were recently defined. The protocol uses X-VIVO 10 serum-free media, supplemented with 450 U/mL of pharmaceutical grade rhuIL-2, and 2.5% allogeneic or autologous human serum or plasma (64). Cells maintained in gas-permeable culture bag systems with regular addition of fresh supplemented media achieve >200-fold expansion in 15–17 days, from a starting population of 6.25 × 10^6^ cells to approximately 1.5 × 10^9^ total cells per 1 L-culture. Patients with solid tumors or leukemia/lymphoma (*n* = 2) were treated with two infusions of escalating doses of NK-92 cells given 48 h apart, with no infusion-related or long-term side effects being observed (63). NK-92 cell doses ranged from 1 × 10^9^ to 1 × 10^10^ cells/m^2^. The dose of 10^10^ cells/m^2^ was considered the maximum expandable cell dose. NK-92 cells persisted *in vivo* for at least 48 h, as shown by Y chromosome-specific PCR in two female patients. Some responses were observed in patients with lung cancer. Only one patient developed anti-HLA antibodies, despite the allogeneic nature of NK-92 cells. NK-92 cells (Neukoplast^®^) will continue to be tested in patients with solid tumors, e.g., Merkel cell cancer and renal cell carcinoma, and with hematological malignancies^1^ (Table 1).

Since several decades, EBV-immortalized B-lymphoblastoid cells (EBV-BLCL) are known to robustly support NK cell *in vitro* expansion and anti-tumor activity (113–115). Escudier and colleagues used 35-Gy-irradiated LAZ 388 EBV-BLCL for the *ex vivo* expansion of NK cells from patients with metastatic renal cell adenocarcinoma. NK cells were initially cultured in V-bottom microplates, at a 4:1 feeder cell to NK-cell ratio, in DMEM medium supplemented with 200 U/mL IL-2. Two to five days before clinical use, NK cells were transferred to Baxter bags, where they received an additional 250 U/ml IL-2 boost. On average, expansion of cultured NK cells was limited to 50-fold after 21 days. However, some clinical responses were observed when autologous NK cells were used as consolidation treatment for patients in partial remission (116). Berg and co-workers described a GMP-compliant protocol involving a 20:1 EBV-BLCL feeder to NK-cell ratio and 500 U/ml IL-2. This system allowed for a 300- to 930-fold NK-cell expansion. EBV-BLCL feeders prevalently drove such an extensive phenomenon, as the use of PBMCs in similar conditions yielded inferior results (69). Based on this protocol, a phase I clinical trial is currently investigating technical feasibility and clinical efficacy of large-scale NK infusions (up to 1 × 10^9^/kg) in cancer patients receiving bortezomib administered with the scope of increasing susceptibility of tumor cells to NK- mediated lysis (117, 118).

K562 engineered to express mbIL-15 and 41BB-ligand (K562–mb15–41BBL) may be used to efficiently propagate NK cells with enhanced anti-leukemia properties. NK cells typically reach a >20-fold expansion after 7 days of co-culture, and a >1,000-fold expansion after 3 weeks, with no concomitant T cell propagation (67, 119). NK cells from patients with MM may also efficiently grow when co-cultured with K562–mb15–41BBL (120). When grown in GMP-compliant gas-permeable static cell culture flasks (G-Rex), as many as 19 billion unmanipulated NK cells can be obtained in 8–10 days starting from 150 million NK cells (93). Importantly, K562–mb15–41BBL cells have been successfully used to expand NK cells transduced with an anti-CD19-BB-ζ CAR, which display enhanced reactivity to CD19^+^ leukemia cells (119). Similar to K562–mb15–41BBL, K562 genetically modified to express mbIL-21, or to co-express the ligand for 41BB and the NKG2D ligand MICA (K562–4-1BBL–mMICA), have been shown to promote large-scale expansion of NK cells with enhanced anti-tumor *in vitro* reactivity (121–123).

### Impact of expansion methods on NK-cell function and homing potential

There are theoretical concerns that extensive *in vitro* expansion may affect the replicative potential and long-term viability of *in vivo-*infused NK cells. For instance, both Fas expression and susceptibility to apoptosis are increased after culture of NK cells with IL-2 or with feeder cells (124). In addition, expanded NK cells down-regulate receptors required for homing into secondary lymphoid organs, such as CCR7, a member of the G protein-coupled receptor family, and CD62L. In line with this, NK cells expanded with genetically modified K562 cells were shown to predominantly express a CD16^+^CD56^+^ phenotype, with no detectable CCR7 (125). To obviate this, NK cells have been cultured with genetically modified, IL-21/CCR7 expressing K562 cells. These culture conditions reportedly resulted into transfer of CCR7 to 80% of expanded NK cells by trogocytosis, a fast, contact-dependent uptake of membrane fragments, and molecules from “donor” to “acceptor” cells (126). CCR7 conferred migratory properties to NK cells by enhancing lymph node homing upon adoptive transfer to athymic nude mice. NAM dose-dependently increases CD62L expression on IL-2/IL-15-stimulated NK cells (82). NK cells expanded with NAM displayed better *in vitro* cytotoxic activity against a variety of tumor cell lines, including leukemia cells, and enhanced homing, as well as *in vivo* persistence in NOD-SCID mice.

Recently, two GMP-grade NK cells products manufactured at different production assistance for cellular therapies (PACT) facilities were evaluated for homing characteristics, i.e., freshly activated (FA)-NK, used by the Minnesota group, and *ex vivo*-expanded (Ex)-NK, developed by the Baylor College of Medicine group (93, 127). Although the two preparations had phenotypic differences, cytotoxicity against NK-sensitive targets was similar. *In vivo* recovery after the infusion of thawed products was lower compared with the infusion of fresh NK cells. Whereas the negative impact of cryopreservation on FA-NK was rescued by overnight culture with IL-2, this strategy was less effective on Ex-NK cells, suggesting the need for optimized cell processing methods (127). NK cells could be detected at day 7 but failed to further expand between day 7 and day 14. Interestingly, higher numbers of functional NK cells with enhanced expression of NKG2A were recovered in mice infused with Ex-NK cells and given IL-15. The homing pattern of the two products was different, with higher numbers of NK cells being detected in the BM of mice given Ex-NK cells and IL-15 compared with Ex-NK cells and IL-2. Conversely, mice receiving FA-NK cells had more NK cells in the spleen when given IL-15. This study emphasizes the importance of continued cytokine stimulation for *ex vivo*-expanded cells, and suggests that differences in the manufacturing process affect *in vivo* homing and clinical efficacy of the NK-cell product.

## Clinical Trials with NK Cells in Hematological Malignancies

### Autologous NK cells

Early clinical studies exploited LAK-based immunotherapy in the autologous setting. One hundred eight patients with refractory metastatic cancer received LAK cells generated from autologous PBMCs incubated with 1,000 U/ml IL-2 for 3–4 days. Systemic high-dose IL-2 was given to support LAK cells *in vivo* (128, 129). Objective tumor regression occurred in 22% of 106 evaluable patients. Median response duration was 10 months for eight patients achieving complete remission (CR). Further prospective studies assessing the therapeutic effects of high-dose IL-2 and LAK cells indicated a possible survival advantage for patients with melanoma treated with LAK cells (130).

Immunotherapy with systemic IL-2 and autologous LAK cells was also given as consolidation treatment after autologous bone marrow transplantation (BMT). Sixteen patients with lymphoma received, 12–14 days post-transplantation, LAK cells generated from PBMCs incubated with IL-2 for 5 days (131). In a similar setting, NK cells obtained prior to transplant and activated with IL-2 for 6 days were infused into 12 patients with advanced cancer and post-BMT pancytopenia (132). Concomitant with NK-cell transfer, sequential high to low-dose systemic IL-2 was also administered for over 90 days. This approach was well tolerated and resulted in the early enhancement of NK-cell activity in four recipients (132).

In general, trials with high-dose systemic IL-2 to support circulating LAK or NK cells were limited by severe and potentially lethal toxicities (e.g., vascular leak syndrome, oliguria, hypotension, myocardial infarction), counterbalancing the beneficial anti-cancer effects of LAK activity (129, 131, 132). On the other hand, chronic low-dose IL-2 treatment was relatively well tolerated (133–135), but unable to activate NK cells as robustly as high-dose *ex vivo* IL-2, or IL-2 at concentrations that engage the intermediate-affinity IL-2 receptor on NK cells (134, 136, 137). Subsequent studies sought to maximize NK-mediated anti-tumor effects. *Ex vivo* IL-2-activated NK-cell infusions were compared with supplemental intravenous IL-2 boluses on days 28 and 35 during daily subcutaneous IL-2 administration in patients with relapsed lymphoma or metastatic breast cancer. Both treatment conditions induced strong NK-cell anti-tumor reactivity, and boosted circulating cytokines, without any consistent impact on clinical outcome compared with matched patients from the Autologous Blood and Marrow Transplant Registry database (138).

The proliferation potential of NK cells isolated from cancer patients may be similar to that of NK cells from healthy donors, reassuring about the feasibility of manufacturing autologous NK-cell products. Although autologous NK cells persist *in vivo* for at least 1 week after infusion, they express lower levels of NKG2D, a key activating receptor, and may necessitate *in vitro* re-activation with IL-2 to lyse tumor targets (68).

Collectively, the analysis of phase II immunotherapy studies with autologous NK cells failed to show efficacy (139). Several factors may have accounted for the disappointing results, including competition with the recipient’s lymphocytes for cytokines and “space”; inhibition of autologous NK cells by self-MHC (30, 140, 141); chronic immunosuppression induced by the tumor on host immunity; and expansion of Treg cells by IL-2 (127). Autologous NK cells are currently being tested in patients with hematological malignancies and solid tumors (NCT00720785; Table 2) (142). In this trial, which is recruiting participants, patients will receive immune suppressive therapy with pentostatin, followed by bortezomib to sensitize tumor cells to NK cytotoxicity (143), escalating doses of autologous NK cells and IL-2.

**Table 2:** **Completed and ongoing clinical trials with NK cells for patients with hematological malignancies are listed**.

Pts.	Diagnosis	NK-cell preparation	Status	Clinical site	Reference(s)/NCI Identifier
10	Children with MRD-negative AML	Conditioning with cyclophosphamide and fludarabine; inhibitory KIR-HLA-mismatched NK cells to reduce relapse risk; 6 doses of 1-million U/m^2^ IL-2 starting on day -1	Published	St Jude Children’s Research Hospital, Memphis, TN, USA	(59)
6	Leukemia, LY	Immunotherapy with NK cells, rituximab + GM-CSF; phase I	Completed (08/12)	M.D. Anderson Cancer Center, Houston, TX USA	NCT00383994
30	Lymphoma	CD56 selection	Published	Duke University, NC, USA	(144) NCT00586690
22	AML, MDS, JMML	Haploidentical donor-derived NK cell infusion and chemotherapy (CY, FLU, IL-2)	Completed (04/14)	St Jude Children’s Research Hospital, Memphis, TN, USA	NCT00640796
13	AL, LY, MY	Allogeneic NK cells post-ABMT; phase I	Completed (06/12)	Tufts Medical Center, Boston, MA, USA	NCT00660166
48	ALL, JMML, AML, MDS, NHL	Haploidentical NK cells after chemotherapy with clofarabine, CY, and etoposide; IL-2; phase I	Completed (03/13)	St Jude Children’s Research Hospital, Memphis, TN, USA	NCT00697671
13	AML	NK cells from haploidentical KIR-ligand mismatched donors after FLU/CY chemotherapy, followed by IL-2; phase I	Published	Univ. of Bologna, Italy	(75)
14	BCP-ALL	Gene-modified NK cells; phase I	Not recruiting	St Jude Children’s Research Hospital, Memphis, TN, USA	NCT00995137
86	Hematological malignancies	NK cells after MRD or MUD HSCT in children with solid tumors and leukemia; phase I	Recruiting	NCI, Bethesda, MD, USA	NCT01287104
13	Hematological malignancies	NK cells and UCBT; phase I	Recruiting	M.D. Anderson Cancer Center, Houston, TX, USA	NCT01619761
90	High-risk AML	Donor NK cells and IL-2 before HSCT with CD34^+^ cells and RIC; phase I/II	Active, not recruiting	Masonic Cancer Center, University of Minnesota	NCT00303667
6	CD20^+^ relapsed NHL or CLL	Allogeneic NK cells; CY, FLU and rituximab followed by IL-2; phase I/II	Published	Masonic Cancer Center, University of Minnesota	(76)
6	Relapsed NHL or CLL	Donor NK cells, rituximab, IL-2 and chemotherapy; phase I/II	Terminated early [failure to meet primary outcome (NK expansion)]	Masonic Cancer Center, University of Minnesota	NCT00625729
47	Hematological malignancies	Donor NK cells after haploidentical HSCT; phase I/II	Completed (02/13)	Asan Medical Center, Seoul, Korea	NCT00823524
10	MM	*In vitro*-expanded haploidentical NK cells; phase I/II	Recruiting	University Hospital, Basel, Switzerland	NCT01040026
15	Hematological malignancies (and solid tumors)	Pre-emptive NK-DLI early after HSCT; phase I/II	Active, not recruiting	University Hospital, Basel, Switzerland	NCT01386619
33	High-risk AML in CR, not eligible for HSCT	CNDO-109-activated allogeneic NK cells; phase I/II	Ongoing; preliminary results (7 patients) presented at 2014 ASH Meeting	United States (multi-center)	NCT01520558 and ref. (77)
30	AL, MDS	HLA-haploidentical NK cells following salvage chemotherapy for patients who have relapsed or persistent leukemia following allogeneic HSCT; phase II	Ongoing, not recruiting	Memorial Sloan-Kettering Cancer Center, NY, USA	NCT00526292
2	Relapsed ALL	Haploidentical NK cells + epratuzumab and low-dose IL-2; phase II	Terminated (slow accrual)	M.D. Anderson Cancer Center, Houston, TX, USA	NCT00941928
					(145)
34	NHL, CLL	Lymphodepleting chemotherapy with rituximab and allogeneic NK cells; phase II	Recruiting	Masonic Cancer Center, University of Minnesota	NCT01181258
43	AML, MDS	NK-cell-based non-myeloablative haploidentical HSCT; phase II	Recruiting	Masonic Cancer Center, University of Minnesota	NCT01370213
6	CML	NK cells and non-myeloablative HSCT; phase II	Completed (11/14)	M.D. Anderson Cancer Center, Houston, TX, USA	NCT01390402
46	MDS	Decitabine and vorinostat conditioning followed NK cell infusion and IL-2; phase II	Recruiting	Masonic Cancer Center, University of Minnesota	NCT01593670
18	Refractory/relapsed AML	Neukoplast™ (NK-92); 1-5 × 10^9^/m^2^; phase I	Recruiting	Conkwest, Inc.	NCT00900809
15	Hematological malignancies	Neukoplast™ (NK-92), 1-5 × 10^9^/m^2^; relapse after autologous HSCT; phase I	Recruiting	University Health Network, Toronto	NCT00990717
16	AL, MDS	NK cells and IL-2 before UCBT; phase II	Terminated (competing study started)	Masonic Cancer Center, University of Minnesota	NCT00354172
18	LY or solid tumors	*Ex vivo*-expanded allogeneic NK cells (MG4101); phase I	Completed (03/13)	Seoul National University Hospital, Korea	NCT01212341
N.A.	Hematological malignancies, solid tumors	Autologous NK cells 24h after treatment with bortezomib; IL-2; phase I	Recruiting	National Heart, Lung, and Blood Institute, MD, USA	NCT00720785

AML, acute myeloid leukemia; MRD, minimal residual disease; ALL, acute lymphoblastic leukemia; LY, lymphoma; MY, multiple myeloma; JMML, juvenile myelomonocytic leukemia; NHL, non-Hodgkin lymphoma; CY, cyclophosphamide; FLU, fludarabine; UCBT, umbilical cord blood transplantation; RIC, reduced intensity conditioning; MRD, matched related donor; MUD, matched unrelated donor; NCI, National Cancer Institute; ASH, American Society of Hematology. *Clinical-grade CTV-1 lysate that primes NK cells *ex vivo;* see main text for further details. N.A., not available. www.clinicaltrials.gov website, last accessed March 2015.

### Allogeneic NK cells

Initial clinical trials on allogeneic T-cell depleted (TCD) haploidentical HSCT for patients with AML showed enhanced NK-mediated cytotoxicity when KIR-HLA class I mismatch occurred (23). In 2005, Miller and co-workers administered haploidentical NK cells in a non-transplantation setting to 43 patients with advanced cancer. Three pharmacological regimens of different intensity were used to prevent immunological rejection (57). After a single leukapheresis, CD3^+^ T cells were depleted under GMP conditions using CD3 microbeads. The TCD product was activated overnight with IL-2 before infusion. NK cells were enriched to 40% on average after processing. The final IL-2-activated product contained an NK-cell dose of 8.5 × 10^6^ cells/kg of recipient’s body weight and a final T-cell dose of 1.75 × 10^5^ cells/kg. A low-intensity immune suppressive regimen was administered on an outpatient basis to the first 17 patients, followed by the infusion of escalating doses of NK cells. Importantly, no dose-limiting toxicity occurred in this patient cohort. Using RT-PCR primers for donor-specific MHC class I alleles, donor cells were shown to persist for 5 days and to comprise <1% of circulating PBMCs, likely due to immune rejection. Alternative low-intensity (fludarabine alone) and high-intensity immune suppressive regimens (high-dose cyclophosphamide and fludarabine) were subsequently used in seven patients with renal cell carcinoma and 19 patients with poor-prognosis AML, respectively. None of the patients given fludarabine alone engrafted with donor NK cells. By contrast, 8 of 15 evaluable patients with AML showed at least 1% engraftment of donor cells after NK-cell infusion. Overall, five patients achieved a morphological CR. Among the four patients with a KIR-ligand mismatch in the graft-versus-host direction, three achieved CR. This was paralleled by the observation that CR was obtained only in 2 of 15 patients without alloreactivity. Finally, IL-15 serum levels were significantly higher in patients receiving higher-intensity, lymphodepleting immune suppression, suggesting that a rise in endogenous IL-15 may be required for the *in vivo* expansion and persistence of infused NK cells.

Subsequent studies suggested the possibility that IL-2 administered after NK infusions also expands Treg cells, potentially interfering with *in vivo* NK-cell proliferation (76). To address this issue, IL-2 diphtheria toxin (IL-2DT) was administered to 15 patients with relapsed/refractory AML, 1 day before the enriched NK product (145). IL-2DT is a recombinant cytotoxic fusion protein composed of the amino acid sequences for diphtheria toxin followed by truncated amino acid sequences for IL-2. IL-2DT reportedly depletes IL-2 receptor α-chain (CD25)-expressing cells, including Treg cells. In this study, three processing methods were used to prepare NK-cell products, including CD3 depletion alone, CD3 depletion followed by CD56 selection, and single-step CD3/CD19 depletion. Higher NK-cell doses were obtained after depletion of CD3^+^ and CD19^+^ cells from a 5-h donor apheresis collection. Among the 42 patients who did not receive host Treg depletion, 21% achieved remissions. By contrast, Treg depletion with IL-2DT resulted in remissions at day 28 for eight of 15 patients (53%). The magnitude of NK-cell expansion was also higher after Treg depletion. The ability of IL-2DT to deplete Treg cells was further supported by reductions in serum IL-35 concentrations 14 days after adoptive transfer. Finally, 7 out of 10 patients (70%) with detectable donor NK cells attained CR by day 28 compared with only 1 of 5 patients (20%) lacking detectable donor NK cells at day 7, suggesting that NK-cell persistence is required for clinical efficacy. Interestingly, this study showed no correlation between achievements of CR- and KIR-ligand mismatch.

Haploidentical, KIR-ligand-mismatched NK cells have been safely infused in elderly patients with high-risk AML, with some evidence of clinical benefit, especially for patients treated in CR or for those with molecular disease relapse (75). Approximately, 40% of the screened patients had a KIR-ligand-mismatched donor, suggesting that this strategy may be applicable to a significant proportion of patients with AML. Another study attempted to exploit KIR-ligand-mismatched NK cells from haploidentical family donors in patients with relapsed MM (74). The apheresis products were TCD and then cultured with IL-2, either overnight or during incubation with anti-CD3 beads. Patients received melphalan and fludarabine as conditioning regimen. After NK-cell infusion, IL-15 levels increased. The response rate (RR) was 50%, with no patient developing GVHD. However, donor chimerism was eventually lost in conjunction with the appearance of host–anti-donor immune responses. The clinical application of allogeneic NK cells to patients with MM is further encouraged by the observation that most MM cell lines are susceptible to NK attack *in vitro*, showing no evidence for HLA class I loss (146).

Resting human NK cells can be primed to kill NK-resistant tumor cells by co-incubation with a clinical-grade lysate of the leukemia cell line CTV-1 (CNDO-109). CNDO-109-activated NK cells remain primed, with no requirement for IL-2 treatment, and can be cryopreserved. The safety, outcome and NK chimerism data from an ongoing phase I/II transitional clinical trial of CNDO-109-NK cells have been recently reported (77). This 3 × 3 dose-escalation phase 1 study was opened in 2013 for patients with high-risk AML in first CR and with no conventional treatment options available. Patients received preparative chemotherapy consisting of cyclophosphamide and fludarabine on study day-6 to -2, followed by a single dose of CNDO-109-activated NK cells on day 0. Patients were given different doses of NK cells (cohort 1 = 3 × 10^5^, cohort 2 = 1 × 10^6^, and cohort 3 = up to 3 × 10^6^ cells/kg). CNDO-109-NK cells were manufactured from a single apheresis collection from HLA-haploidentical-related donors. NK cells were isolated with anti-CD56 microbeads and co-incubated overnight with CNDO-109 lysate under GMP conditions (Coronado Biosciences)^2^. Residual T-cell contamination (defined as <10^4^ cells/kg patient body weight) was considered a safety criterion for lot release. Seven eligible patients were enrolled. No infusion-related toxicities or adverse events directly attributed to NK therapy were observed, including GVHD. Patients experienced transient myelosuppression lasting approximately 2 weeks. Three patients relapsed early post-treatment (average time to relapse from CR1 being 104 days). In five of seven evaluable patients, persistence of activated donor NK cells was observed from day 7 post-infusion to as late as day 56 in one patient. The comparison of donor and patient endogenous NK cells showed a mature activated phenotype of donor NK cells. Two of the three patients evaluated had persistence of low levels of activated autologous NK cells (~10–20% of circulating NK cells), exceeding the numbers circulating pre-treatment. This observation may indicate that NK-cell therapy induces endogenous NK activation and enhances innate immunity to AML in the absence of exogenous cytokine administration. When the study was published in abstract form, four of the seven patients enrolled were relapse-free.

Natural killer cells have also been administered pre-emptively to patients with high-risk cancer, after TCD haploidentical HSCT (73). Sixteen patients were treated in a prospective phase II study with purified NK cells on day 3, 40, and 100 after HSCT. The median dose of NK cells was 12.1 × 10^6^/kg. With a median follow-up of approximately 6 years, 4 out of 16 patients were alive. The four patients who developed acute GVHD had received >0.5 × 10^5^/kg contaminating T cells. Compared with a historical cohort of patients treated with haploidentical HSCT without NK donor lymphocyte infusions (DLIs), NK cells apparently exerted no effect on disease relapse.

#### Trials in Children and Young Adolescents

A landmark study from St. Jude Children’s Hospital (NKAML) has shown the safety, feasibility, and engraftment potential of haploidentical NK cells for children with favorable and intermediate-risk AML (59). Patients received a mild conditioning regimen, consisting of cyclophosphamide (60 mg/kg on day-7) and fludarabine (25 mg/m^2^/day from day-6 to -2), followed by KIR-HLA-mismatched NK cells (median number of cells infused 29 × 10^6^/kg) and six doses of IL-2 (1 × 10^6^ U/m^2^). Donor PBMCs obtained by leukapheresis were depleted of CD3^+^ T cells and then enriched for CD56^+^ cells. The manipulated product contained a very low number of contaminating B cells and T cells. The resulting NK population was infused fresh, without any incubation with IL-2. All patients were in CR, as shown by minimal residual disease status. NK infusions were well tolerated, with no GVHD observed. NK-cell engraftment was transient in all patients, with a median peak NK-cell chimerism of 7%. One patient had prolonged NK engraftment with 2% of donor NK cells being detected at day 189, in association with delayed neutrophil and platelet engraftment. With a median follow-up of approximately 32 months, all patients remained in remission. The 2-year EFS estimate was 100%.

The therapeutic potential of NK cells has also been tested in young adolescents. A two-step T-cell depletion strategy with a final CD56 enrichment step was pursued to isolate NK cells from steady-state leukapheresis collections (147). Purified NK cells were expanded for 2 weeks in X-VIVO 10 medium containing 10% human AB serum and 1,000 U/ml IL-2. This procedure resulted in a median 95% NK-cell purity, 99% viability, and enhanced cytotoxicity against the K562 line as well as primary leukemic blasts obtained from patients. T-cell contamination was negligible (<0.1%). Three children with multiply relapsed ALL or AML were treated with IL-2-stimulated NK cells after haploidentical HSCT. Directed KIR mismatches in the GVL direction were present in all three cases. Remission was achieved in all cases, although patients ultimately died of infectious complications or disease relapse. Another study in children and young adolescents with ultra-high-risk solid tumors has shown that donor-derived NK cells activated with IL-15 and 41BBL can be safely administered after HLA-matched TCD HSCT (148). NK cells displayed potent killing capacity. However, five of nine transplant recipients developed acute GVHD, with grade III GVHD being observed in three patients. The unexpected occurrence of GVHD in this report may be attributed to timing of NK-cell infusion, lack of post-transplantation immune suppression or activation of NK cells that were expanded on IL-15-secreting feeder cells (149). The observation that GVHD developed in all four patients given unrelated donor transplants compared with one of five patients given related donor transplants points to a T-cell-driven mechanism, mediated by minor antigens and accentuated by the infused NK cells.

### *In vivo* targeting of NK cells with antibodies

IPH2101 is a first-in-class, non-depleting human IgG_4_ mAb directed against inhibitory KIRs, and functions by blocking inhibitory KIR–ligand interactions, leading to restored or augmented NK-cell function against tumor cells. A phase I trial of IPH2101 (#NCT00552396) was conducted in 32 patients with relapsed/refractory MM (150). IPH2101 was given intravenously every 28 days in sevn dose-escalated cohorts (0.0003–3 mg/kg) for up to four cycles. This study identified doses of IPH2101, which conferred KIR2D occupancy *in vivo*, with no concomitant dose-limiting toxicity or identification of a maximally tolerated dose (MTD). With one exception, adverse events were mild and transient and mainly consisted of self-limited infusion reactions. Although IPH2101 enhanced *ex vivo* patient-derived NK-cell cytotoxicity against MM, no objective responses were observed. Another phase I study of escalating doses of IPH2101 in 23 elderly patients with AML in first CR showed a correlation between IPH2101 exposure and KIR occupancy (151). Adverse events were mild and transient, consisting mainly of infusion syndrome and erythema. The study drug did not affect the numbers and distribution of lymphocyte subsets and NK cell receptor expression. At the highest dose levels, TNF-α and MIP-1β serum concentrations, as well as CD69 expression on NK cells, transiently increased. Overall and relapse-free survival compared favorably to reports in other patient populations with similar characteristics (151).

An immunization study of transgenic mice bearing human immunoglobulin loci with different combinations of KIR2DLs has recently led to the identification of the 1-7F9 mAb, based on binding to soluble, recombinant KIR2DL1, -2, and -3 by ELISA (152). The 1-7F9 antibodies bind to human NK cells, γδ^+^ T cells, and CD8^+^ T cells, consistent with KIR expression patterns. Using *in vitro* assays, 1-7F9 blocked the binding of HLA class I to inhibitory KIR2DLs, augmenting NK cell-mediated killing of HLA-C-expressing targets. 1-7F9 also enhanced the cytotoxicity of NK cells from an HLA-C-matched donor against AML blasts. Pre-treatment with 1-7F9 of patient-derived NK cells translated into a two- to threefold increase in cytotoxicity against autologous AML cells. Finally, studies in NOD-SCID mice showed that 1-7F9 potentiates NK-mediated killing and promotes mice survival compared with co-infusions of NK cells and AML blasts alone (152).

### Bi-specific and tri-specific antibodies

A novel class of therapeutics uses either all or part of the antibody structure to deliver enhanced effector activity to the tumor site. The fusion of two (bi-specific) or three (tri-specific) portions of the fragment of antigen-binding (Fab) region of a traditional antibody yields reagents with high level of antigen specificity and cross-links tumor antigens with potent immune effectors. Bi-specific killer engagers (BiKEs) are constructed with a single-chain Fv against CD16 and a single-chain Fv against a tumor-associated antigen. The mechanisms by which BiKEs and TriKEs potentiate NK effector functions include intracellular calcium mobilization through direct CD16 signaling (153). Co-culture of reagent-treated resting NK cells with Raji targets also translates into increased NK-cell degranulation, target cell death, and NK production of IFN-γ, TNF-α, GM-CSF, IL-8, MIP-1α, and RANTES.

Fully humanized CD16 × 33 BiKEs have been shown to trigger NK-cell activation *in vitro* against CD33^+^ AML cell lines and primary refractory CD33^+^ AML targets (154). Combination treatment with BiKEs and ADAM17 inhibitor to prevent CD16 shedding further enhanced NK-cell function. BiKEs were also effective at activating NK cells from recipients of double UCB transplantation. BiKEs enhance degranulation and cytokine production by NK cells derived from patients with myelodysplastic syndromes and cultured with CD33^+^ AML cell lines, irrespective of disease stage and age stratum (155). A potential drawback of this approach is the relatively short half-life of the antibody constructs, with limited trafficking to the tumor site.

### Other approaches to target NK cells *In vivo*

A first in-human trial of *Escherichia coli*-produced rhIL-15 has been recently published (156). The primary objectives of this single-institution, open-label, non-randomized 3 + 3 design, phase I dose-escalation study were to assess safety, dose-limiting toxicity, and MTD of rhIL-15 given as intravenous bolus at 3.0, 1.0, and 0.3 μg/kg/day for 12 consecutive days to patients with metastatic malignant melanoma or renal cancer. Reductions in the number of lymphocyte subsets were evident within 20 min of the infusion of IL-15, with the most dramatic decline being observed with NK cells, γδ^+^ T cells and CD8^+^ memory T cells. The acute efflux of CD8^+^ cells from the circulation was most pronounced for transitional and effector memory subsets. An influx of NK cells to the blood was detected by 4 h, followed by a normalization of cell counts by 2–3 days. The initial influx of cells mainly resulted from redistribution, because no evidence of proliferation was observed until 48 h. CD8^+^ cells showed evidence of activation, including proliferation and increased expression of CD38 and HLA-DR. Dose-limiting toxicities in patients receiving 3.0 and 1.0 μg/kg/day included grade 3 hypotension, thrombocytopenia, and elevations of ALT and AST, resulting in 0.3 μg/kg per day being determined the MTD. Common cytokine-related adverse events, including fever, rigors, and hypotension occurred much less frequently in patients treated with the 0.3 μg/kg dose level. Cytokines such as IFN-γ, TNF-α, IL-6, and IL-8 increased up to 50-fold in patient serum after rhIL-15 administration. Overall, there were no clinical responses in this study, with stable disease being recorded as a best response. However, five patients manifested a decrease between 10 and 30% in their marker lesions, with two of these patients experiencing clearing of lung lesions (156). This study proves that rhIL-15 administration, as an intravenous bolus dose, is associated with clinical toxicities due to marked cytokine secretion. The authors initiated a dose-escalation trial of continuous intravenous infusion of rhIL-15 to patients with metastatic malignancies. Furthermore, the Cancer Immunotherapy Trials Network (CITN^3^) is conducting a phase I dose-escalation trial of subcutaneous rhIL-15 administered 5 days per week for 2 weeks.

## Current Manufacturing Practices for CIK Cells

**Figure 2:**
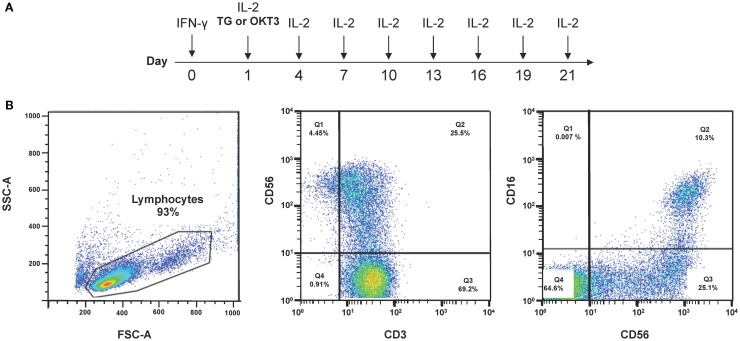
**Protocols to generate CIK cells from PBMCs**. **(A)** Current protocols used to differentiate CIK cells from PBMCs rely on IFN-γ, anti-CD3 mAbs or thymoglobulin (TG), and IL-2. **(B)** A representative experiment depicts the phenotype of CIK cells after 3-week culture under the above cytokine conditions.

Current protocols to differentiate CIK cells are based on the combination of 1,000 IU/ml IFN-γ on day 1 of culture followed 24 h later by 50 ng/ml OKT3 and 300 IU/ml IL-2 (157). After 21–28 days, CD3^+^CD56^+^ cells, derived from CD3^+^CD56^−^ cells, acquire cytotoxicity against various tumor cell targets, including AML, chronic myeloid leukemia, and B-cell and T-cell lymphoma. The expression of CD56 on CIK cells is thought to result primarily from IFN-γ priming with subsequent IL-12 production from monocytes. Recently, a GMP-grade protocol (ITG2) that incorporates thymoglobulin^®^(TG) was used to prepare CIK cells (Figure 2; Table 3) (45). TG is a purified, pasteurized preparation of polyclonal γ immunoglobulin raised in rabbits against human thymocytes (45). TG expanded CIK cells more efficiently than the anti-CD3 mAb when provided to clinical-grade cultures in combination with IFN-γ and IL-2. Higher levels of NKG2D, NKp46-triggering receptor, and killer-like immunoglobulin receptors KIR2DL1 and KIR2DL2/DL3 were detected on CIK cells differentiated with TG compared with those obtained with αCD3 antibodies. CIK cells were capable of lysing tumor cell targets in an MHC-unrestricted manner and released high quantities of bioactive IL-12p40. The use of the ITG2 protocol was not associated with the emergence of Treg cells *in vitro*, thus reassuring against the infusion of excessive numbers of tumor-suppressive T-cell populations.

**Table 3:** **Completed and ongoing clinical trials with CIK cells for hematological malignancies are listed**.

Pts.	Diagnosis	CIK cell preparation	Status	Clinical site	Reference(s)/NCI Identifier
11	AML, HL, CML, ALL, MDS	LK or PB; 1,000 U/ml IFN-γ + OKT3 + IL-2 for 20–26 d; median number of total allogeneic CIK cells 2.4 × 10^6^/kg (7.2–87.4); phase I	Published	Italy	(157)
5	AL	UCB washout after UCB transplantation	Published	Italy	(162)
9	DLBCL	Autologous PB; 2,000 U/ml IFN-γ + OKT3 + IL-2	Published	China	(163)
10	Lymphoma (*n* = 2)	1,000 U/ml IFN-γ + OKT3 + IL-2; CIK cells then transfected with pCEP-IL-2-plasmid	Published	Germany	(164)
1	Plasmocytoma	Autologous CIK cells; monthly for 21 courses	Published	China	(165)
12	Lymphoma (*n* = 6)	LK or PB; median 28 × 10^9^ (range, 6–61) CIK cells per patient	Published	Italy	(166)
41	MDS/MPD	Post-transplantation infusion of allogeneic CIK cells as consolidation therapy; phase II	Recruiting	Stanford University, USA	NCT01392989
17	AML/MDS	Autologous CIK cells after HSCT or in patients unfit for standard curative chemotherapy; phase I/II	Completed	Singapore General Hospital	NCT00394381
11	CML	Adoptive immunotherapy in patients receiving standard drug therapy; phase II	Completed	Singapore General Hospital	NCT00815321
20	Hematological malignancies	Allogeneic CIK cells as post-HSCT immunotherapy; phase I	Active, not recruiting	NIH, US	NCT00185757
20	Hematological malignancies	Allogeneic CIK cells for relapse after allogeneic HSCT; phase I/II	Recruiting	Singapore General Hospital	NCT00460694

*PBMC, peripheral blood mononuclear cells; AL, acute leukemia; DLBCL, diffuse large B-cell lymphoma; LK, leukapheresis; CML, chronic myeloid leukemia; MDS, Myelodysplastic syndromes; MPD, myeloproliferative disorders; d, day; UCB, umbilical cord blood; SCGM, stem cell growth medium; MM, multiple myeloma. www.clinicaltrials.gov website last accessed March 2015*.

The administration of bulk CIK cells is not associated with GVHD in mice, even after sequential infusions (158). The same study also showed that bulk CIK cells are more effective than selected CD56^+^ CIK cells or CIK cells depleted of potentially alloreactive αβ^+^ CIK cells.

Several investigators have successfully obtained CIK cells from UCB units. The washouts of UCB units may yield approximately 500 × 10^6^ CIK cells after a standardized 21-day expansion culture (159). Compared with PB-derived CIK cells, UCB CIK cells demonstrate lower immunogenicity and higher proliferative capacity and anti-tumor activity in pre-clinical models of cancer (160). Interestingly, UCB-derived CIK cells released higher amounts of IL-2 and IFN-γ and expressed higher levels of CCR6 and CCR7, pointing to a better ability to traffic to tumor sites and secondary lymphoid organs. CIK cells differentiated from PB and UCB also differ in receptor expression and in cytotoxic activity against ALL cells, suggesting that the source of CIK cells may impact on therapeutic efficacy (161).

## Clinical Trials with CIK Cells

An International Registry on CIK Cells (IRCC) has been established with the aim of reporting results from clinical trials centered on adoptively transferred CIK cells. In the first IRCC publication (167), eleven clinical trials with autologous or allogeneic CIK cells were identified, with 426 patients enrolled. Most trials included male patients with hepatocellular carcinoma, gastric cancer, and relapsed lymphoma. A clinical response was reported in 384 patients, who received up to 40 infusions of CIK cells. The total RR was 24% and a decrease of tumor volume was documented in three patients. DFS rates were significantly higher in patients treated with CIK cells than in a control group without CIK treatment. An update published in 2014 (168) enlists the results obtained in 2,729 patients from 45 phase I/II studies. A total of 1,520 patients with 22 different tumor entities were treated with CIK cells, either alone or in combination with chemotherapy. Allogeneic CIK cells were employed in the majority of the trials (41 out of 45). The number of CIK cells infused varied among the different trials, averaging 7.7 × 10^9^ cells. Data on patient survival were available for 19 out of 45 trials; 15 of these 19 trials were paired, as they also included control patients receiving none or standard therapy alone. Overall, a beneficial effect of CIK cells emerged in patients with hepatocellular carcinoma, renal cell carcinoma, non-small cell lung cancer (NSCLC), colorectal carcinoma, and breast cancer. High numbers of CIK cells at time of infusion were associated with a better prognosis. Quality of life was also improved in four of the five trials for which data were disclosed. Ancillary biological data were provided in 23 studies. The absolute numbers of T cells, as well as serum IFN-γ, were increased after immunotherapy compared with baseline. Some studies also reported a decrease of blood Treg cells after CIK infusions. Immunotherapy was generally well tolerated, with fever occurring in 41% of cases and headache and fatigue reported in 30% of cases (168). Mild GVHD was observed in seven of 52 patients treated with allogeneic CIK cells and was responsive to steroid therapy. Based on these findings, the IRCC currently recommends the use of at least 10 × 10^9^ CIK cells with at least 30% CD3^+^CD56^+^ cells per infusion, every 2–4 weeks, for at least six times. The IRCC website^4^ (last accessed March 2015) lists 1,787 patients treated with CIK cells, mostly for hepatocellular carcinoma, gastric cancer, ovarian cancer, renal cell cancer, MM, and NSCLC.

China has the largest population of patients with malignant tumors and is the country where most clinical trials with CIK cells were conducted (169). Using the VIP database of Chinese scientific and technological journals^5^, 24 articles were selected. Information on the total number of CIK cells used was available in 16 studies and ranged from 6 × 10^6^ to 1.5 × 10^10^ in one single treatment course. As far as hematological malignancies are concerned, 1 study specifically dealt with non-Hodgkin’s disease (12 patients) and 4 studies with AML (51 patients). Only 14 out of 24 studies contained details about clinical outcome. Of the reported 563 patients, 40 had a CR, 126 had a partial response, 125 had a minimal response, 135 had stable disease, and 58 had progressive disease. The remaining 76 patients did not reach an objective response. The overall RR was 51.7% (291/563) (169). Information on patient outcome was provided in 10 studies. Four studies reported a 1-year OS rate of 72.5%, six studies reported 2-year OS rate of 66.3%, one study reported 3-year overall RR of 75.5%, and two studies reported 5-year OS rate of 38.2%.

A phase I study of allogeneic CIK cells has been conducted in 11 patients with hematological malignancies relapsing after allogeneic HSCT (157). Six patients had received other salvage treatments before CIK administration, including DLIs, without significant clinical response. The median number of CIK cells infused was 12.4 × 10^6^/kg, with no infusion-related toxicities recorded. Acute GVHD occurred in four patients 30 days after the last CIK infusion, and progressed into extensive chronic GVHD in two cases. Disease progression and death were observed in six patients. One patient had stable disease, one experienced hematologic improvement, and three obtained complete responses. The same authors differentiated CIK cells from UCB samples and administered them to five patients relapsed after UCB transplantation (162). In three patients, chemotherapy had been given before CIK administration to reduce disease burden. Infusions of a median of 1.5 × 10^6^/kg CIK cells were provided early after leukemia relapse. Some clinical response was observed in one patient who also developed acute intestinal GVHD. The remaining four patients experienced disease progression and died. GVHD was managed with steroids and mesenchymal stromal cells but the patient ultimately succumbed to leukemia relapse (162).

In another study (170), CIK cells were generated for 24 patients with advanced-stage hematological malignancies, mostly from allogeneic donors, either under steady-state conditions or after stem cell mobilization. Overall, 55 infusions were given to 16 patients at doses ranging from 10 to 200 × 10^6^ CD3^+^ cells/kg. Notably, the proportion of the CD3^+^CD56^+^ subset was higher in CIK cultures derived from patients than in those differentiated from healthy donors. The median expansion of CD3^+^ T cells and CD3^+^CD56^+^ NK-like T-cells was 9.33-fold and 27.77-fold, respectively. Responses attributable to CIK infusions were documented in five patients, including two with ALL, two with Hodgkin disease, and one with AML. In five patients, the response to CIK cells could not be assessed as salvage chemotherapy or dasatinib was concomitantly administered. Acute GVHD occurred in three patients and was manageable. For three of the six patients failing to respond, leukemia cells were available for *in vitro* killing assays, which showed only modest cytotoxicity of donor CIK cells against tumor targets. Overall, this study in advanced-stage hematological malignancies suggests that CIK administration may translate into some clinical efficacy with a modest toxicity and low incidence and severity of GVHD.

Finally, autologous immune effector cells generated by TG, IFN-γ, and IL-2 (ITG2) can be safely administered to patients with advanced and/or refractory solid tumors (Figure 2) (171). After 2–3 weeks in culture, a median of 4.65 × 10^6^ immune effectors/kg of recipient’s body weight was infused intravenously without observing any toxicity. One patient with advanced melanoma died because of disease progression before the infusion of CIK cells. The target dose of at least 2.5 × 10^6^ CIK cells/kg of recipient’s body weight was reached in four out of five evaluable patients. The median survival was 4.5 months (range 1–13) from the first infusion of CIK cells (171).

## Closing Remarks

Although the field of NK cell and CIK cell-based immunotherapy is rapidly advancing, some pre-clinical and clinical issues need to be clarified before this immunotherapy approach is widely offered to patients with hematological malignancies and solid tumors (172).

Cell therapy products enriched for NK cells using CD3 depletion and CD56 selection contain variable percentages of monocytes. It is now established that both monocytes and monocyte-derived DCs can support NK-cell proliferation and function (173). This implies that different NK-cell manufacturing protocols may affect the cellular composition of the final products and impact on NK-cell function. Also, the optimal number of NK cells to be infused remains to be determined. Patients reportedly tolerate target doses of 2 × 10^7^ NK cells/kg without any serious side effect (78). This number of NK cells can be routinely obtained from a 1-day large-volume leukapheresis. However, it is possible that higher doses and/or multiple infusions of NK cells may be required for optimal clinical efficacy. Whether NK cells may exert their beneficial effects pre-emptively in patients with disease remission, or rather in the context of HSCT warrants particular attention (73). In this respect, KIR ligand mismatch is expected to mediate a more powerful effect in T-cell-depleted HSCT, since alloreactive T cells reportedly blunt NK reactivity (37, 174). Finally, recent discoveries on NK-mediated allorecognition will guide the choice of the optimal NK-cell donor in order to maximally exploit the anti-tumor effect (33). The isolation of single KIR^+^ NK cells under GMP conditions is feasible and yields clinically applicable numbers of alloreactive NK cells (65).

Current clinical evidence also points to CIK cells as a potentially useful immunotherapy approach for cancer patients. Similar to other forms of immunotherapy, the infusion of CIK cells may be more efficacious at disease stages where the tumor burden is relatively low or in an adjuvant setting, rather than for advanced disease (175).

## Conflict of Interest Statement

The authors declare that the research was conducted in the absence of any commercial or financial relationships that could be construed as a potential conflict of interest.

## Footnotes

^1^http://www.conkwest.com/nk-92

^2^http://www.coronadobiosciences.com/research-development/cndo-109.cfm

^3^http://citninfo.org/citn-science/clinical-studies.html

^4^http://www.cik-info.org/index.php?kat=tableclinicaltrials

^5^http://oldweb.cqvip.com
